# Health-Seeking Behavior Regarding Coughs in Urban Slums in Lagos, Nigeria

**DOI:** 10.3390/medicines10070038

**Published:** 2023-06-26

**Authors:** Victor Abiola Adepoju, Olanrewaju Oladimeji, Olusola Daniel Sokoya

**Affiliations:** 1 Department of HIV and Infectious Diseases, Jhpiego–An Affiliate of Johns Hopkins University, Abuja 900108, Nigeria; 2 Department of Public Health, Faculty of Health Sciences, Walter Sisulu University, Mthatha 5099, Eastern Cape, South Africa; 3 Department of Epidemiology and Biostatistics, School of Public Health, Sefako Makgatho Health Sciences University, Pretoria 0208, South Africa; 4 Lagos State Tuberculosis, Buruli Ulcer and Leprosy Control Program, Lagos 100001, Nigeria

**Keywords:** tuberculosis, health-seeking behavior, urban, slum, patent proprietary medicine vendor, world TB Day, cough, Nigeria, active case finding, outreaches

## Abstract

Background: TB is a major cause of morbidity and mortality, with slum residents being disproportionately affected. This study aimed to assess health-seeking behavior among adult residents of slum communities presenting with coughs in Lagos, Nigeria. Methods: A community-based, cross-sectional study was conducted across six urban slums in Nigeria as part of community outreaches to mark World TB Day. A structured, pretested questionnaire was used to capture relevant sociodemographic details and questions regarding symptoms of coughs and related symptoms as well as care-seeking behavior. Data were explored, analyzed, and presented using descriptive statistics. Results: A total of 632 respondents participated in this study. The majority were 25–34 years old (24.7%), male (65.8%), Christian (55.7%), married (73.7%), with secondary education (37.8%), with 3–4 persons per household (41%) and with 1–2 persons per room (44.5%). In total, 26.6% had had a cough for two weeks or more and were considered as presumptive TB patients. Overall, 37.2% of respondents with a cough visited patent proprietary medicine vendors (PPMVs) as the first port of call. Good health-seeking behavior was exhibited by only 36.2% of respondents. In total, 38.9% delayed seeking care from a health facility (government or private) more than one month after the onset of symptoms. None of the factors included in the multivariate analysis showed a significant association with good health-seeking behavior (i.e., visiting government or private hospitals/clinics). Conclusions: The poor health-seeking behavior, delay in seeking TB care and preference for PPMVs emphasizes the need for National tuberculosis programs (NTPs) to further engage these informal providers in TB prevention, diagnosis and treatment services in urban slum communities.

## 1. Introduction

Tuberculosis (TB) is a global public health problem and contributes significantly to the disease burden in Nigeria. Nigeria ranks among the top eight countries with the highest number of new TB cases [1]. In urban slums, the incidence of TB is higher compared to other communities given the high poverty levels, limited access to healthcare, and other risk factors [2]. However, urban slums are often overlooked in TB control programs. Prevention, early detection, and treatment are crucial for effective TB control, as delayed diagnosis and treatment can lead to continued transmission and the development of advanced TB cases [3]. Moreover, health-seeking behavior among individuals with coughs has received limited attention in Nigeria, particularly in urban slum communities, where the risk of TB transmission is heightened. However, it is important to focus on the specific symptom of interest, which is a cough, as it is a common presenting symptom in individuals with respiratory conditions, including tuberculosis. Delayed presentation to healthcare facilities, misdiagnosis, and patient-related delays contribute to gaps in case notification and missed opportunities for early TB diagnosis and treatment [4,5,6]. Several studies have explored health-seeking behavior among individuals with coughs, providing more insights into the associated factors and predictors [4,5,6,7,8]. Understanding the factors influencing health-seeking behavior in this population is crucial for the identification of gaps in TB control programs and can also help in designing targeted interventions. Furthermore, studies conducted in urban slums have highlighted the burden of TB and emphasized the need for active case finding [2,9]. Additionally, investigations in different settings have examined health-seeking behavior among individuals with presumptive TB, emphasizing the importance of early diagnosis and treatment [9,10,11].

Cough is a common symptom in respiratory conditions, including tuberculosis (TB). It is one of the cardinal symptoms for the diagnosis of respiratory diseases, including TB, if it lasts up to 2 weeks or more. The persistent nature of a cough, especially when lasting for more than two weeks, warrants attention, as it may be indicative of underlying health concerns. However, the health-seeking behavior of individuals with coughs, particularly in urban slum settings, remains understudied. Understanding the factors that influence the health-seeking behavior specifically related to coughs is essential for improving early detection, timely treatment, and effective TB control strategies.

Therefore, this study aims to investigate health-seeking behavior among urban slum dwellers with coughs in six slum communities in Lagos, Nigeria. By examining the specific context of urban slums, this study will contribute to the understanding of health-seeking behavior in a high-risk population, providing valuable insights for the development of strategies to improve TB case detection and access to timely treatment.

## 2. Materials and Methods

### 2.1. Study Design

This was a community-based, cross-sectional study. The study took place from 1 to 31 March 2017 as part of the World TB Day activities. Community-based TB case finding (outreach) was carried out across six slum communities in Local Government Areas (LGAs) with high TB prevalence in Lagos, Nigeria.

### 2.2. Study Setting

The study took place in Lagos, Nigeria, an urban city in southwestern Nigeria. Lagos has a population of approximately 14,862,000 as of 2021, with 20 Local Government Areas (LGAs) and 57 Local Council Development Areas (LCDAs) [12]. Within these LGAs, there are about 11 private and government health facilities serving an estimated population of 50,109 individuals in the slum communities, predominantly consisting of individuals from disadvantaged backgrounds.

The recruitment of subjects for the study was carefully conducted through community entry, mobilization, and sensitization efforts until a sample size of 632 participants was reached. The selection of slum communities was based on their official classification as urban poor communities and their high burden of tuberculosis (TB) and HIV. These communities exhibited densely populated and congested residential conditions, with a significant mix of migrants and temporary settlers commonly found in typical urban slums.

The choice of sample size was determined by accounting for practical considerations, feasibility, and the available resources. The study was designed to provide valuable insights into the dynamics and factors influencing health-seeking behavior within the specific slum communities under study. The focus of the research was to gain a comprehensive understanding of health-seeking behavior in these specific settings, rather than making population-level generalizations. The selected LGAs, including Ikeja, Ojo, Ifako Ijaiye, Apapa, and Ajeromi-Ifelodun, were identified based on their high burden of TB and HIV. These LGAs were categorized as priority areas for HIV epidemic control and the scale-up of related interventions, including testing, counseling, and laboratory support for antiretroviral therapy supplies. These locations were considered hotspots where the ongoing transmission of tuberculosis and other comorbidities could be high.

### 2.3. Definition of Terms

‘Seeking care’ is defined as the action taken by an individual with presumptive TB to seek relief for symptoms.

Cough: A cough is a reflex action of the respiratory system that helps to clear the airways of irritants, mucus, and foreign substances. There are different types of coughs, and they can vary in duration and severity and may or may not be accompanied by other symptoms.

Presumptive TB: An ‘individual with presumptive TB’ was defined as a person who had been coughing for ≥2 weeks at the time of the survey.

‘Good health-seeking behavior’: This was defined as a visit to either a government or private/mission health facility after the onset of TB symptoms.

‘Poor health-seeking behavior’: This referred to any other care decision, e.g., doing nothing, self-medication, a visit to PPMVs, or a visit to traditional healers.

Delayed health-seeking behavior: This referred to making the decision to seek care 30 days after the onset of symptoms of a chronic cough.

### 2.4. Study Size

The total sample size was 625, with an average of 104 respondents per community.

### 2.5. Sampling Technique

Six slum communities were purposively selected across 5 LGAs because of the high burden of tuberculosis in Lagos, Nigeria. A multistage sampling technique was adopted to ensure the representation of diverse slum communities. In the first stage, three streets were randomly selected in each of the six study communities. Subsequently, systematic random sampling was employed to select houses within each street. From each selected house, one household was chosen using simple random sampling. Eligible respondents were then consecutively selected per household for mobilization on the day of the outreach until the sample size was reached for that community. In cases where there were no eligible household members, there was unwillingness to participate, or residents were not available, the next household was approached.

### 2.6. Participants

The final study size of 632 participants was purposively determined to achieve sufficient statistical power for the analysis. The average number of subjects per community was 104. Although the sampling technique was purposive, it was driven by the aim of capturing a representative sample from diverse slum communities with a high tuberculosis burden. While a stratified random sampling approach could have provided more precise estimates, the chosen sampling strategy was practical and feasible given the resource constraints and logistical challenges encountered in conducting research within slum settings.

### 2.7. Inclusion and Exclusion Criteria

Participants were included in the study if they were 18 years of age or older, had a history of a cough, and had resided in the slum community for at least 3 months. The inclusion criteria aimed to capture individuals who were likely to have experienced the local health-seeking dynamics and challenges within the slum communities.

### 2.8. Data Sources/Measurement

We used a 16-item questionnaire to ask about the cough status and health-seeking behavior of the participants. The questionnaire consisted of two parts. The first part included the participants’ socio-demographic information including age, sex, occupation, marital status, religion, ethnicity, average monthly income, educational level, and number of persons sleeping in a room. In the second part, health-seeking behavior towards cough symptoms was assessed by asking the following questions: Yes or no questions were asked regarding symptoms of a cough. If the participants answered “Yes”, they were further asked about the duration of the cough and whether they had accompanying symptoms like weight loss, night sweats, fevers, coughing out blood, and loss of appetite, as well as the duration of any of the positive symptoms. We categorized the duration of cough symptoms into two categories: <2 weeks and >2 weeks. In the present study, we defined health-seeking behavior as health-seeking decisions and the choice of medical facility (government or private). Participants were asked questions on where they went to after the onset of symptoms of a cough (a chemist, government hospital, private hospital, self-medication, or a traditional healer) to understand their healthcare-seeking decision. We also asked questions on how long after the onset of the cough they sought care and the reasons for not seeking care, among others. Good health-seeking behavior was defined as a visit to either a government or private/mission health facility/clinic after the onset of TB symptoms, while poor health-seeking behavior referred to a visit to any other setting.

### 2.9. Data Collection

Data were collected using a structured interviewer-administered questionnaire. The tool was pre-tested in a similar community outside of the study sites, and relevant corrections were made. The survey collected information on socio-demographic characteristics (age, sex, income, occupation, marital status, household occupancy, smoking, alcohol, etc.) and health-seeking behavior towards cough symptoms. Face-to-face interviews were conducted by twelve trained research assistants (two per outreach location) with higher education and fluency in the local Nigerian languages. To ensure the quality of the data collected, the research assistants carrying out the data collection were also supervised by more senior research scientists assigned to each of the research locations. After community health workers initially identified eligible participants, face-to-face interviews were conducted in a private setting, specifically under a canopy, following the assessment of participants’ vital signs. Respondents’ interviews took an average of 20 min to complete. Subsequently, sputum samples were taken from individuals who presented with cough symptoms, and these were taken to the laboratory via sputum transporters for TB testing. The results were mostly available to participants within 24 h. Participants who could not collect the results were followed up within 48 h via their phone number collected during the interviews.

### 2.10. Data Analysis

Data analysis was performed with Statistical Package for Social Sciences (SPSS) version 21. The time taken to seek care after the onset of symptoms was classified into 4 categories: within 2 weeks, 2 weeks–30 days, >30–60 days, and more than 60 days. A duration less than 30 days was coded as prompt health-seeking, and a duration over 30 days was coded as delayed health-seeking behavior. Similarly, seeking care in either government or private/mission facilities was coded as good health-seeking behavior, while seeking care elsewhere was coded as poor health-seeking behavior. Categorical variables including socio-demographic characteristics were presented using descriptive statistics such as frequencies and percentages.

### 2.11. Ethical Consideration

The study was approved by the Health Research and Ethics Committee of Lagos State University Teaching Hospital (LREC. 06/10/828). Respondents’ confidentiality was also maintained by not using identifiers. An explanation of the purpose, the benefits, and the possible risks of the study was also provided. To anonymize study participants, code numbers rather than personal identifiers were used. Written informed consent was obtained from the respondents prior to the administration of the questionnaires.

## 3. Results

Table 1 shows the sociodemographic characteristics of the respondents. The study achieved a 100% response rate, and a total of 632 respondents participated in this study. The majority were in the age group of 25–34 years (24.7%), male (65.8%), Christian (55.7%), married (73.7%), with secondary education (37.8%), with 3–4 persons per household (41%), and with 1–2 persons per room (44.5%). Table 2 shows the various symptoms of TB experienced by the respondents. The majority of the respondents with symptoms had a cough (34.2%), followed by a fever (23.6%), and the symptom least commonly reported was hemoptysis (3.2%). Table 3 shows the health-seeking behavior of respondents after they had symptoms of a cough. Of the 632 respondents, 26.6% had a cough for two weeks or more and were considered as presumptive TB patients. The majority of the respondents who had cough initially went to a patent proprietary medicine vendor (37.2%), while 20.8% went to a government hospital to seek medical attention. Good health-seeking behavior was exhibited by 36.2%, and poor health-seeking behavior was exhibited by 63.8%. Table 4 shows the determinants of health-seeking behavior among patients with presumptive/confirmed TB. None of the factors included in the analysis showed a significant association with good health-seeking behavior (i.e., visiting government or private hospitals/clinics).

**Figure 1:**
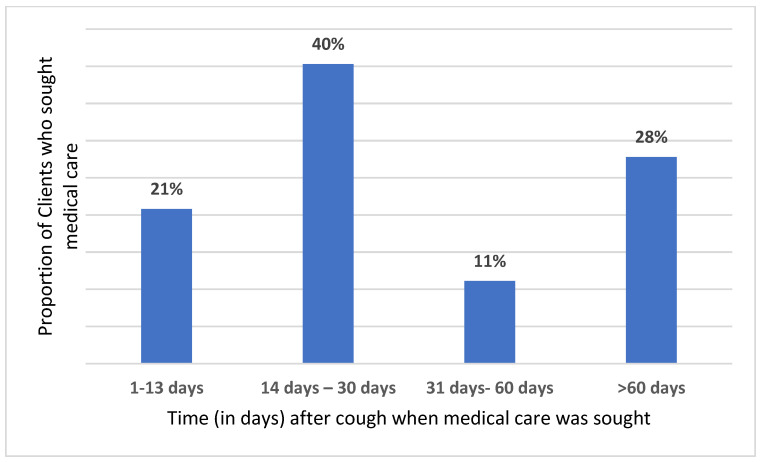
Time after cough when respondents sought medical care.

**Figure 2:**
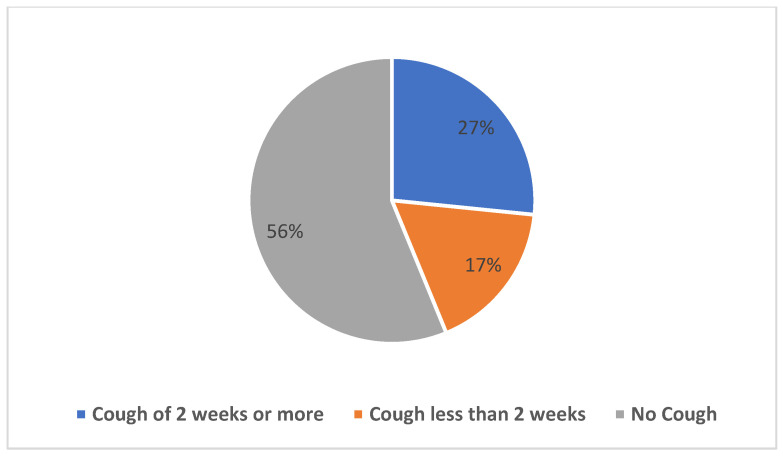
Duration of cough among respondents, n = 632.

Figure 1 shows that the majority of respondents that sought care after the onset of cough did so between 2 weeks and 30 days (40.3%), followed by respondents who acted after 60 days (27.8%). Figure 2 shows the duration of coughs among the respondents, with the majority (26.6%) having had a cough duration of 2 weeks or more (presumptive TB) during presentation.

## 4. Discussion

This study sought to determine the health-seeking behavior among presumptive TB patients in urban slums in Lagos, Nigeria. A cough was the most common symptom reported by study participants, with about one-quarter of respondents classified as presumptive TB patients. This is consistent with other studies in Nigeria [9] but was higher than studies in Ethiopia, Zambia, Tanzania, and Bangladesh [2,3,10,12,13]. A possible explanation is that the studies in Ethiopia, Tanzania, and Zambia focused on the whole population, while the current study specifically assessed the population in urban slums.

Our findings suggest that there was poor health-seeking behavior among the study participants. Only about a third had good health-seeking behavior and sought care from qualified personnel (hospitals). This is consistent with local and international studies [14,15]. The rate of good health-seeking behavior in government and private/mission hospitals was 36.2% in our study, which is higher than finding from Vietnam, where only 24% of presumptive TB clients sought care in hospital settings [16], but lower than 81% who visited public and private hospitals in Uganda [17] and 60.9% who visited modern health facilities (defined as health centers, health posts, hospitals, or private clinics) in Ethiopia as the first point of care after the onset of a cough [10]. The Vietnamese study specifically targeted patients who sought care at referral hospitals and exhibited a tendency to utilize multiple healthcare systems. As a result, these patients had a higher number of prior hospital visits. [16] A major problem with the Ethiopian study was the possibility of a patient classifying a pharmacy visit as a visit to a modern health facility. This was not clear from the survey options. A qualitative study identified lack of financial resources, stigma, and pill burden as reasons for not seeking care for symptoms of a cough among community dwellers in Uganda [18]. However, our study findings suggest that the independent variables may not play a significant role in determining whether individuals seek care from qualified healthcare providers for their TB symptoms. However, it is important to note that other patient-related factors, socio-cultural drivers, hygiene practices, cultural beliefs, and gender roles may still influence health-seeking behavior but were not fully examined in this study.

Furthermore, about two-fifths delayed seeking care for more than 30 days after the onset of TB symptoms in our study. This is similar to another study in Lusaka, Zambia [19], but lower than 56.3% of patients who delayed seeking care for over 30 days after the onset of symptoms in South Africa [20]. A meta-analysis by Getnet and colleagues reported a delay in seeking care in 42% of presumptive patients [21].

It was clear from our study that the majority and almost two-fifths of patients with cough symptoms preferred visiting patent proprietary medicine vendors after the onset of symptoms, which is higher than the 13.5% who were found to do so in Uganda [17], lower than the 48.9% found to do so in Labanon [22] but comparable to the 32% who sought care from informal providers in South Africa [20]. The accessibility and affordability of treatment at PPMVs may explain this preference [23]. However, it should be noted that previous studies have highlighted concerns regarding the quality of tuberculosis (TB) evaluation provided by drug outlets, as well as poorer treatment outcomes and the inappropriate use of drugs [24,25]. Hence, while patients may seek care for a cough at PPMVs for reasons of convenience and affordability, caution should be exercised regarding the accuracy of TB evaluation in these settings. PPMVs also lack the medical expertise and trained human resources needed to be able to diagnose and manage TB patients. Depending on the category, some PPMVs are nurses and other allied health professionals with basic health trainings and qualifications. Therefore, TB control programs and strategies should intensify efforts to engage PPMVs in TB control programs. Providing training to PPMVs on cough identification and evaluation and procedures for referral can help reduce delays in diagnosis and treatment and facilitate the identification of “missing TB cases”, thereby increasing case notification rates [26]. National tuberculosis programs should strengthen the adoption of the World Health Organization’s Public-Private Mix (PPM) roadmap, expand training, and incorporate more PPMVs into the TB control program to bridge knowledge and practice gaps [27,28].

### Study Limitations

The study results were derived from the Nigerian urban slum population but could also be generalized to other populations with a similar slum context. One limitation of the study is that participant responses were based on self-reported data, which may be subject to recall bias. Additionally, the survey did not include inquiries about whether patients’ samples were collected for tuberculosis (TB) diagnosis, thus limiting the comprehensive understanding of the entire TB diagnostic pathway. In addition, due to the limited available resources, the study only focused on pulmonary TB with coughs being used to pre-screen slum residents. Other limitations of this study include the definitions of health-seeking behavior, which did not fully capture a patient-centered perspective by not considering the relative acceptability and availability of TB services in slum communities. Additionally, the data collection timeframe in 2017 may not reflect potential changes in health-seeking behaviors, including the impact of the COVID-19 pandemic. Future research should address these limitations and delve deeper into the determinants of health-seeking behavior in TB-affected slum communities.

## 5. Conclusions

Our findings highlight that only slightly over one-third of respondents with coughs sought care in government or private hospitals. Among those who sought care, a significant proportion experienced delays in seeking treatment, with about 38% delaying care-seeking for one month or longer after the onset of a cough. Additionally, the majority of individuals seeking care for coughs patronized informal patent proprietary medicine vendors. This situation mirrors the healthcare landscape in Nigeria and many African countries, where the predominantly accessible healthcare providers are PPMVs, and modern facilities are scarce, inadequate, and often located far from the slum population. Addressing this challenge requires the National TB Program to explore and scale up engagement with informal providers who are not currently affiliated with the program. Training and mentoring initiatives should be implemented to enhance TB case finding, treatment, and overall quality of care in slum communities. It is crucial for future studies on health-seeking behavior to delve into patient-related factors and socio-cultural drivers of poor health-seeking behavior, particularly examining how hygiene practices, behaviors, cultural beliefs, and gender roles influence health-seeking behavior in slum communities. These efforts will contribute to improving TB control strategies and address the multi-factorial barriers that impede timely and appropriate care-seeking behaviors among individuals with prolonged cough symptoms.

## Figures and Tables

**Table 1:** Sociodemographic characteristics of respondents *N* = 632.

Characteristics	Frequency	Percentage
**Age (years)**		
<15	8	1.3
15–24	33	5.2
25–34	156	24.7
35–44	151	23.9
45–54	138	21.8
55–64	82	13.0
>65	64	10.1
**Sex**		
Male	416	65.8
Female	216	34.2
**Religion**		
Christianity	352	55.7
Islam	277	43.8
Traditional religion	3	0.5
**Ethnicity**		
Yoruba	469	74.2
Ibo	69	10.9
Hausa/Fulani	44	7.0
Other	50	7.9
**Marital Status**		
Single	91	14.4
Married	466	73.7
Separated/divorced	23	3.7
Widowed	52	8.2
**Education**		
No formal education	132	20.9
Primary	170	26.9
Secondary	239	37.8
Tertiary	91	14.4
**Average no of household**		
**1–2**	99	15.7
**3–4**	259	41.0
**5–6**	180	28.5
**7–8**	51	8.1
**8–10**	16	2.5
**>10**	27	4.3
**No of persons per room**		
**1–2**	281	44.5
**3–4**	243	38.4
**5–6**	90	14.2
**>6**	18	2.9

**Table 2:** Symptoms of tuberculosis experienced by respondents (*N* = 632).

Symptoms	Frequency	Percentage
Cough	216	34.2
Weight loss	112	17.7
Night sweats	123	19.5
Fever	149	23.6
Hemoptysis	20	3.2
Loss of appetite	72	11.4

**Table 3:** Health-seeking behaviors of respondents after respondents had symptoms of cough.

Health-Seeking Behavior	Frequency	Percentage
Did nothing	15	8.8
Self-medication/home remedy	26	15.5
Private hospital	25	15.0
Government hospital	35	20.8
Patent medicine store	62	37.2
Mission hospital	1	0.4
Traditional healer	4	2.2
Overall health-seeking behavior (*n* = 226)		
Good health-seeking behavior	82	36.3
Poor health-seeking behavior	144	63.7

**Table 4:** Determinants of good health-seeking behavior among patients with presumptive/confirmed TB.

Variable	B (Regression Coefficient)	S.E. (Standard Error)	Wald (Wald Chi-Square Test)	df (Degrees of Freedom)	Sig. (Significance)	aOR (Adjusted Odds Ratio)	95% C.I. (Confidence Interval) Lower	95% C.I. (Confidence Interval) Upper
Hx of Contact with TB patient	−0.112	0.401	0.078	1	0.78	0.894	0.407	1.964
Smoking	−0.481	0.72	0.446	1	0.504	0.618	0.151	2.536
Knowledge of TB spread	−0.345	0.339	1.035	1	0.309	0.708	0.364	1.377
Knowledge of TB symptoms	−0.429	0.333	1.667	1	0.197	0.651	0.339	1.249
Education	−0.255	0.454	0.315	1	0.575	0.775	0.319	1.886
Income level	0	0	0.17	1	0.68	1	1	1
Religion	−0.283	0.324	0.766	1	0.382	0.753	0.399	1.421
Sex	0.303	0.333	0.827	1	0.363	1.354	0.705	2.6
BMI (Kg/meter square)	−0.011	0.022	0.251	1	0.616	0.989	0.948	1.032
Age	0.018	0.011	2.753	1	0.097	1.018	0.997	1.039
Constant	−0.102	1.019	0.01	1	0.92	0.903		

BMI = Body Mass Index.

## Data Availability

The data presented in this study are available on request from the corresponding author. The data are not publicly available due to privacy restrictions.

## Abbreviations

AIDS	Acquired Immunodeficiency Syndrome
ART	Anti-Retroviral Therapy
DR-TB	Drug-Resistant Tuberculosis
DSTB	Drug-Susceptible Tuberculosis
HIV	Human Immunodeficiency Virus
LCDA	Local Council Development Area
LGA	Local Government Area
LSTBLCP	Lagos State Tuberculosis and Leprosy Control Program
LTFU	Lost to Follow Up
NTBLCP	National Tuberculosis and Leprosy Control Program
NTP	National Tuberculosis Program
PPMV	Patent Proprietary Medicine Vendors
SPSS	Statistical Package for Social Sciences
TB	Tuberculosis
WHO	World Health Organization
